# Comprehensive serum metabolomics and network analysis to reveal the mechanism of gypenosides in treating lung cancer and enhancing the pharmacological effects of cisplatin

**DOI:** 10.3389/fphar.2022.1070948

**Published:** 2022-12-01

**Authors:** Yan-Shuang Qi, Man-Yu Xiao, Peng Xie, Jin-Bo Xie, Mei Guo, Fang-Fang Li, Xiang-Lan Piao

**Affiliations:** School of Pharmacy, Minzu University of China, Beijing, China

**Keywords:** *Gynostemma pentaphyllum*, gypenosides, cisplatin, metabolomics, lung cancer, network analysis

## Abstract

Gypenosides (GYP) exerted anticancer activity against various cancers. However, the mechanism of GYP against lung cancer (LC) *in vivo* remains unclear. This study aims to reveal the potential mechanism of GYP against LC and enhancing cisplatin efficacy using a comprehensive analysis of metabolomics, network analysis. Pharmacodynamic results showed that GYP inhibited tumor growth, reduced tumor volume and tumor weight, and alleviated pathological symptoms in Lewis tumor-bearing mice, and GYP could enhance the anti-LC effects of cisplatin. Using serum metabolomics methods, 53 metabolites were found to be significantly altered in the model group, and the levels of 23 biomarkers were significantly restored after GYP treatment. GYP-related metabolic pathways involved six pathways, including alpha-linolenic acid metabolism, glutathione metabolism, sphingolipid metabolism, glycerophospholipid metabolism, tryptophan metabolism, and primary bile acid biosynthesis. 57 genes associated with differential metabolites of GYP recovery and 7 genes of 11 saponins of GYP against LC were screened by network analysis, the STRING database was used to find the association between 57 genes and 7 genes, and a compound-intersection gene-metabolite related gene-metabolite-pathway network was constructed, and STAT3, MAPK14, EGFR and TYMS might be the crucial targets of GYP against LC. Western blot results showed that GYP restored the levels of STA3, MAPK14, EGFR, and TYMS in the model group, and GYP also restored the levels of STAT3 and MAPK14 in the cisplatin group, indicating that GYP might exert anti-LC effects and enhance the pharmacological effects of cisplatin through MAPK14/STAT3 signaling pathway. Our method revealed the effect and mechanism of GYP on LC and the pharmacological effects of GYP-enhanced chemotherapeutic agent cisplatin, which provided some reference for the development of anti-cancer drugs.

## 1 Introduction

*Gynostemma pentaphyllum* (Thunb.) Makino is an herbaceous vine of Cucurbitaceae (Kim et al., 2022a), *G. pentaphyllum* is widely used as herb or tea in Asia (Li et al., 2022). *G. pentaphyllum* contains flavonoids, saponins, polysaccharides and other substances. Gypenosides (GYP) is the main bioactive components in *G. pentaphyllum*, which has the effects of anti-oxidation, lowering blood lipid, lowering blood glucose and anti-cancer (Kim et al., 2022b). In Zhuang medicine (Liang and Zhong, 2005) and traditional Chinese medicine theory (Zhao et al., 2006), *G. pentaphyllum* was reported to be able to inhibit the growth of tumors. Lung cancer is the main cause of cancer-related death and one of the most common malignant tumors in the world (Li et al., 2021). Cisplatin is a platinum-based anticancer drug. It is the first-line drug for clinical chemotherapy of lung cancer, but cisplatin has side effects, cisplatin has been reported to be more effective in combination with traditional Chinese medicine than cisplatin alone (Qiu et al., 2021).

Metabolomics involves the systematic analysis of metabolites produced in cells, body fluids, tissues or organs under drug or pathological conditions (Schmidt et al., 2021). Generally, two techniques are used for metabolic analysis, namely, nuclear magnetic resonance (NMR) spectroscopy and mass spectrometry (Tayanloo-Beik et al., 2020). Metabolomics technology is now widely used to measure the response of endogenous metabolites to the whole body and find biomarkers of many cancers (Yang et al., 2020). Metabolomics plays a crucial role in biomarker discovery and mechanistic interpretation of lung cancer, providing a basis for biomarker screening, early diagnosis and staging of LC (Noreldeen et al., 2020). Metabolomics is a promising area in cancer research, which is divided into non-targeted and targeted metabolomics, and the purpose of non-targeted approaches is to obtain as many metabolites as possible from a range of biological samples (Cheung et al., 2019).

It is reported that GYP had anticancer effects on colorectal cancer, leukemia, oral cancer, hepatocellular carcinoma, bladder cancer, lung cancer and renal cell cancer (Ahmad et al., 2019), (Liu et al., 2021).

**FIGURE 1:**
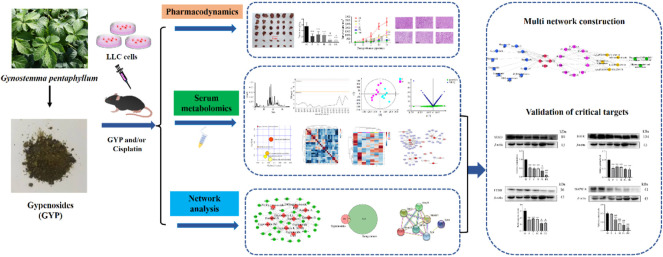
The flow chart of this study.

In our previous study, we explored the mechanism of GYP on A549 cells using network pharmacology and metabolomics (Qi et al., 2021). In this study, we established a Lewis lung cancer mouse model, and then used a combination of serum metabolomics and network analysis to discover GYP-related metabolite markers, and clarified the anticancer mechanism of GYP and enhanced chemotherapeutic drugs pharmacological effects (Figure 1).

## 2 Materials and methods

### 2.1 Chemicals and reagents

Gypenosides (GYP) were provided by our own laboratory (Qi et al., 2021); Cisplatin was purchased from TargetMol (United States). *β*-actin (Zsgb-Bio, TA-09, 1:1000), STAT3 (Proteintech, 60199-1-Ig, 1:2000), TYMS (Proteintech, 15047-1-AP, 1:1000), MAPK14 (Proteintech, 66234-1-Ig, 1:2000), EGFR (Proteintech, 66455-1-Ig, 1:10000).

### 2.2 Animals and cell lines

The Lewis lung carcinoma cell (LLC) line was bought from the National infrastructure of Cell Line Resource (Bejing, China) and cultured in DMEM medium (Gibco) in a humidified atmosphere (5% CO_2_, 37°C). Pathogen free male C57BL/6N mice (18–20 g) were purchased from Beijing Vital River Laboratory Animal Technology Co., Ltd. (Beijing, China, approval number: SCXK-2016-0006).

### 2.3 Cell viability assay

Cell viability was evaluated by MTT assay. For each experiment, 1 × 10^4^ cells were seeded in each well of 96 well plates. Then the cells were attached to the wall after 24 h and used with different concentrations of GYP (0, 120, 250, 500 μg/ml) for 24 h. The OD values were detected at 490 nm using a microplate reader. There were 3 replicates in each group.

### 2.4 Mouse xenograft models and drug administration

The mice were housed in an animal room (24 ± 2°C, 60 ± 5% relative humidity) set to a 12 h dark/light cycle. Mice were fed water and standard laboratory food to adapt to the environment for 7 days before the experiment. After one week, forty-two mice were used to build an LLC tumor-bearing mice model of LLC by subcutaneous injection with 0.1 ml LLC (2 × 10^7^/ml) cells. After the model was constructed successfully, mice were randomly divided into 7 groups, including control group [C], model group [M], cisplatin group [P] (cisplatin, 4 mg/kg), GYP low-dose group [L] (GYP, 40 mg/kg), GYP high-dose group [H] (GYP, 80 mg/kg), cisplatin + low-dose GYP group [PL] (cisplatin, 4 mg/kg; GYP, 40 mg/kg) and cisplatin + high-dose GYP group [PH] (cisplatin, 4 mg/kg; GYP, 80 mg/kg) (n = 6 each). GYP (dissolved in PBS, 8 mg/ml, 16 mg/ml) was intraperitoneally injected into the mice once a day at the dose of 0.1 ml/20 g (dose volume/body weight) for 14 days. Cisplatin (dissolved in PBS, 0.8 mg/ml) was intraperitoneally injected into the mice every other day at the dose of 0.1 ml/20 g (dose volume/body weight) for 7 days. The mice in the control group and model group were all given PBS at the equal volume. The body weights of mice were weighed and recorded every 2 days. The sarcoma sizes were measured and recorded every 3 days, and the length and width of the sarcoma were measured using a vernier caliper, tumor volume was calculated as follows: $tumor\;size=tumor\;length\times {\left(tumor\;width\right)}^{2}/2$ (Wu et al., 2018). Mice were sacrificed on day 15 after successful tumor inoculation, and the blood were collected. Tumor tissues were further removed and weighed, half of which were fixed with 4% paraformaldehyde for HE staining, and the other half were snap-frozen with liquid nitrogen and placed in a -80°C freezer for future use.

### 2.5 Hematoxylin-eosin staining

The tumor tissues of each mouse were fixed in 4% paraformaldehyde and embedded in paraffin, and then tumor tissues were cut into 4 μm sections and stained with hematoxylin and eosin (HE).

### 2.6 Serum metabolomics analysis

#### 2.6.1 Serum collection and preparation

The preparation and pretreatment of serum samples and QC samples were adjusted appropriately according to references (Cai et al., 2022). Mice were fasted in advance for 12 h, and blood was collected in 1.5 ml eppendorf (EP) tubes by retro-orbital bleeding after anesthesia, and then the blood was centrifuged (12000 r/min) for 20 min to collect serum samples. 10 μL of each sample was mixed to collect quality control (QC) samples. 100 μL serum samples were mixed with 400 μL acetonitrile to precipitate proteins, vortexed to pellet completely, and centrifuged at 12,000 r/min and 4°C for 20 min. 450 μL of the supernatant was drawn with a pipette and placed in a centrifugal concentrator for concentration and drying. 80% acetonitrile-water (v/v) was then added for reconstitution and vortexed for 2 min before UPLC-MS analysis.

#### 2.6.2 UPLC-Q-TOF/MS condition

A Waters ACQUITY UPLC HSST3 column (1.8 μm, 2.1 × 100 mm) was used for chromatographic separation, and the mobile phase consisted of 0.1% formic acid aqueous solution (phase A) and 0.1% formic acidacetonitrile (phase B) at a flow rate of 0.2 ml/min. Gradient elution conditions were properly adjust according to literature (Yao et al., 2022), as follows: 0–2 min, 99%–99% A; 2–3 min, 99%–65% A; 3–25 min, 60%–1% A; 25–30 min, 1% A; 30–30 min, 1%–99% A; 30–40 min, 99%–99% A. The total chromatographic run time was 40 min, the injection volume was 5 μL, and the column temperature was 35°C.

The mass spectrometer is a Q/TOF-MS system equipped with an electrospray ionization (ESI) source (Time of Flight ™ 6600, AB SCIEX, United States). Nebulizer gas (GS 1), heater gas (GS 2) and Curtain gas was 40 psi, 40 psi, 35 psi, respectively; the collision energy (CE), declustering potential (DP) and the collision energy spread (CES) were respectively 25/- 25 eV, 80/- 80 V and (±) 12.5 eV; The m/z scan range of TOF MS and IDA MS/MS was 100–1500 and 50-1000, respectively. The ion source temperature was 450°C. After LC- (±) ESI-MS in two ion modes was collected, MSConvert software was used to convert Wiff format files to mzXML format files, then XCMS software is used to obtain a two-dimensional data array including mass to charge ratio (m/z), retention time (RT) and peak area.

#### 2.6.3 Multivariate statistical analysis

The data matrices of two ion modes were imported into SIMCA software (Swedish Umetrics version 14.0) for unsupervised principal component analysis (PCA), validation plot analysis, supervised orthogonal partial least squares discriminant analysis (OPLS-DA) and VIP + S plot analysis, respectively. All QC samples were subjected to Hotelling’s T2 region to test the stability of the instrument. Variable importance in prediction (VIP) > 1.0, combined with an absolute value of *p*corr greater than 0.5 as well as the *p*-value of *t*-test < 0.05, was used to seek differential metabolites between the control group [C] and the model group [M]. The relative contents of differential metabolites between the 7 groups were compared to seek GYP-related differential metabolites.

#### 2.6.4 Determination of potential biomarker, metabolic pathway analysis, correlation analysis and heatmap analysis

HMDB database (https://hmdb.ca/) and METIN database (https://metlin.scripps.edu/) were used to identify the differential metabolites between the control group and model group, and then the relative contents of different metabolites in 7 groups were compared by GraphPad Prism 9 software for exploring the biological effect of GYP on lung cancer, then the 23 metabolites recalled by GYP were imported into MetaboAnalyst 5.0 database (https://www.metaboanalyst.ca/) for analysing metabolic pathway, pearson correlation analysis and heatmap analysis.

### 2.7 Screening for the intersection of compounds targets and disease targets

We obtained GYP-related targets from SwissTargetPrediction database (http://swisstargetprediction.ch/). To deeply dissect the anti-LC effects of GYP on LLC mice, lung cancer associated genes were obtained from National Center for Biotechnology Information database (https://www.ncbi.nlm.nih.gov/) and OMIM database (https://omim.org/). Then the intersection genes of compounds targets and disease targets were exported into STRING database (https://cn.string-db.org/) and Cytoscape_v 3.9.0 software for Protein–Protein Interaction (PPI) network construction.

### 2.8 Multi network construction

In order to investigate how GYP exerts its anti-lung cancer effects by regulating metabolites and metabolic pathways as well as enhancing the anti-lung cancer effects of chemotherapeutic agents, a compound-intersection genes-metabolite related gene-metabolite-pathway network was constructed.

### 2.9 Western blot analysis

Total protein was extracted from tumor tissues, total protein concentration was determined with a BCA kit (Beyotime), then SDS-PAGE electrophoresis was used to separate the same amounts of samples, then the protein was transferred to PVDF membrane, and then the primary antibody (*β*-actin, STAT3, TYMS, MAPK14, EGFR) and secondary antibody were incubated for 2 h respectively, then the PVDF membranes were detected by ECL (Beyotime). The bands were quantitatively analyzed by ImageJ software.

### 2.10 Statistical analysis

All experiments were reported as mean ± SD. One-way analysis of variance (ANOVA) was used for comparing differences among the control group [C], model group [M], cisplatin group [P], GYP low-dose group [L], GYP high-dose group [H], cisplatin + low-dose GYP group [PL], cisplatin + high-dose GYP group [PH]. A *p*-value < 0.05 was considered statistically significant.

## 3 Results

### 3.1 GYP inhibited the viability of LLC cells

**FIGURE 2:**
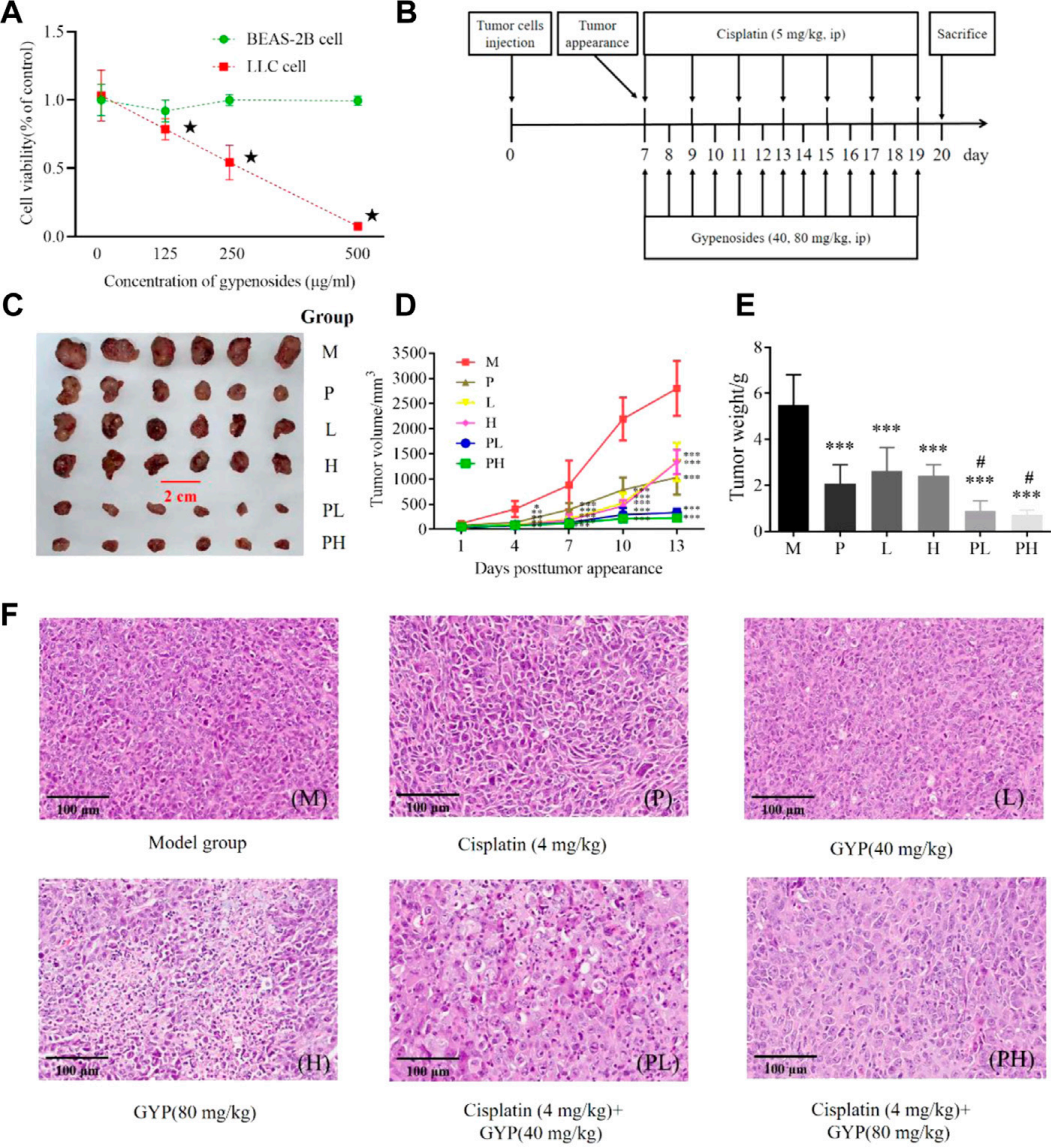
GYP suppressed the growth of xenografted LLC tumors and enhanced cisplatin efficacy in C57BL/6 mice. The cell viability of GYP against LLC cells and BEAS-2B cells, vs. 0 mg/ml GYP, ^★^ *p* < 0.001 **(A)**; Administration procedures in animal experiment **(B)**; Tumor visualization of 6 groups **(C)**; The tumor volumes **(D)** and tumor weights **(E)** of 6 tumor-bearing group (M: model group, P: cisplatin group, L: low-dose GYP group, H: high-dose GYP group, PL: cisplatin + low-dose GYP group, PH: cisplatin + high-dose GYP group; vs. Control group, *p < 0.05, **p < 0.01, ***p < 0.001; vs. Model group, ^#^ *p* < 0.05); The histopathology sections (H&E, magnification = ×40, 50 μm) of tumor of 6 groups (F).

According to Figure 2A, the cell viability was 97.55%, 45.21%, and 15.32% after treated with 125, 250, and 500 μg/ml GYP, respectively. GYP could suppress the proliferation of LLC cells in a dose-dependent manner, and GYP had no significant effect on the activity of BEAS-2B cells within the maximum dose of 500 μg/ml of GYP administered. The IC_50_ value of GYP on LLC cells was 272.5 μg/ml.

### 3.2 GYP decreased the tumor volume and tumor weight of the model group and cisplatin group

The anti-cancer effect of GYP *in vivo* was then conducted on Lewis tumor-bearing mice (Figures 2B–E). Compared with M group, the tumor volume of other 5 groups (P, L, H, PL and PH group) significantly decreased, of which PL group and PH group decreased more (Figure 2C). Compared with M group, the tumor weight of P group, L group, H group, PL group and PH group significantly decreased, of which PL group and PH group decreased more (Figures 2D,E). Thus, GYP could suppress sarcoma growth and enhance the antitumor efficacy of cisplatin.

Histopathological analysis of tumor tissue suggested that the tumor cells in the model [M] group were closely arranged, with obvious nucleoli and diffuse distribution. In the cisplatin + GYP groups (PL and PH group), compared with the single GYP treatment groups (L and H group) and the single cisplatin treatment group (P group), the area with unclear boundary and nuclear disappearance is larger, indicating that GYP had anticancer effect and could enhance the pharmacological effects of cisplatin (Figure 2F).

### 3.3 Metabolomic analysis

**FIGURE 3:**
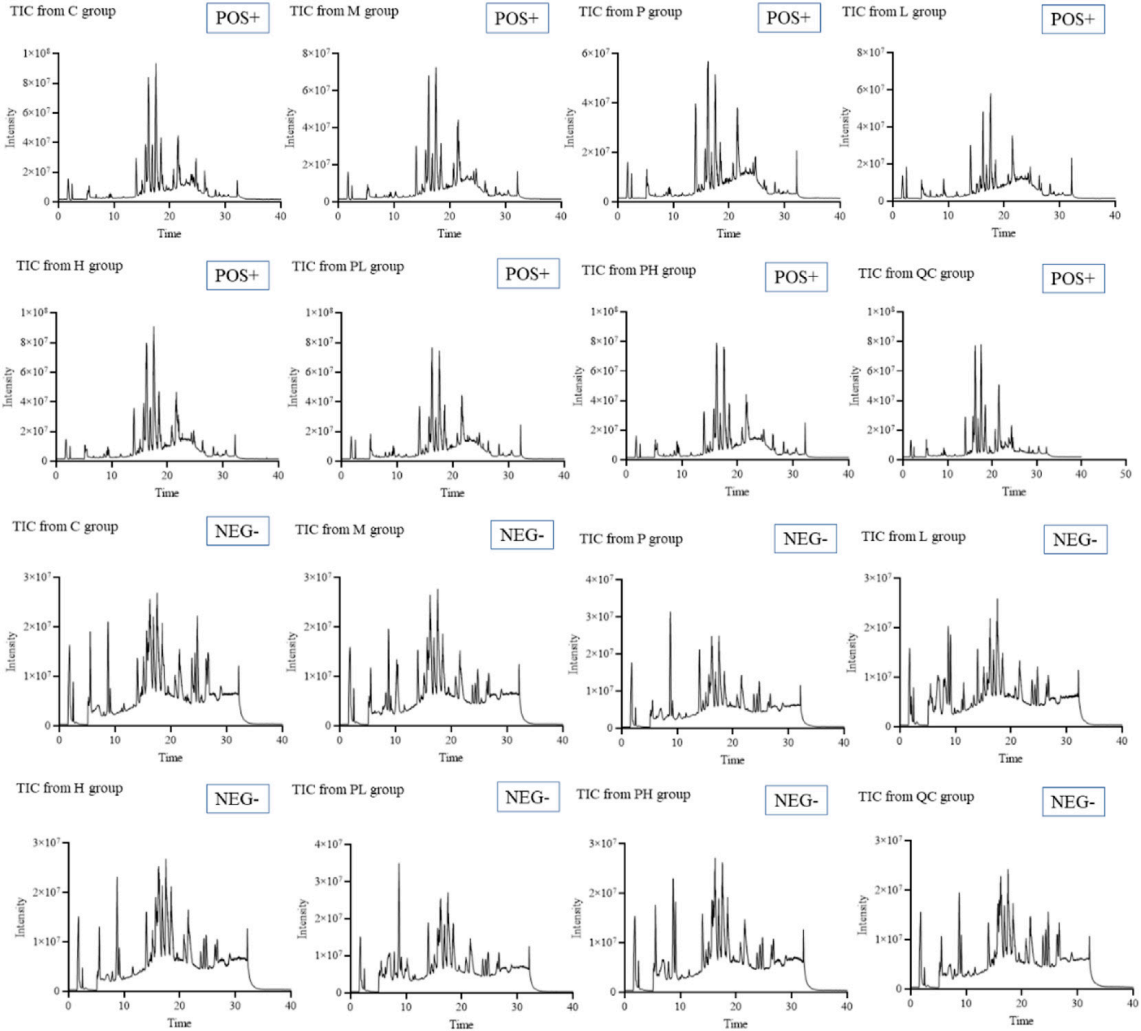
Total ions chromatographs (TICs) of control [C], model [M], cisplatin [P], GYP low-dose [L], GYP high-dose [H], cisplatin + low-dose GYP [PL] and cisplatin + high-dose GYP [pH] in the positive model (POS +) and negative model (NEG -).

The total ion chromatograms (TICs) (Figure 3) were obtained from QC, control [C], model [M], cisplatin [P], GYP low-dose [L], GYP high-dose [H], cisplatin + low-dose GYP [PL], cisplatin + high-dose GYP [PH] group using UPLC-Q-TOF/MS conditions. Endogenous metabolites with molecular weight less than 1000 Da could be well separated within 40 min. A total 27807 and 27665 ions were respectively extracted from the positive and negative datasets.

**FIGURE 4:**
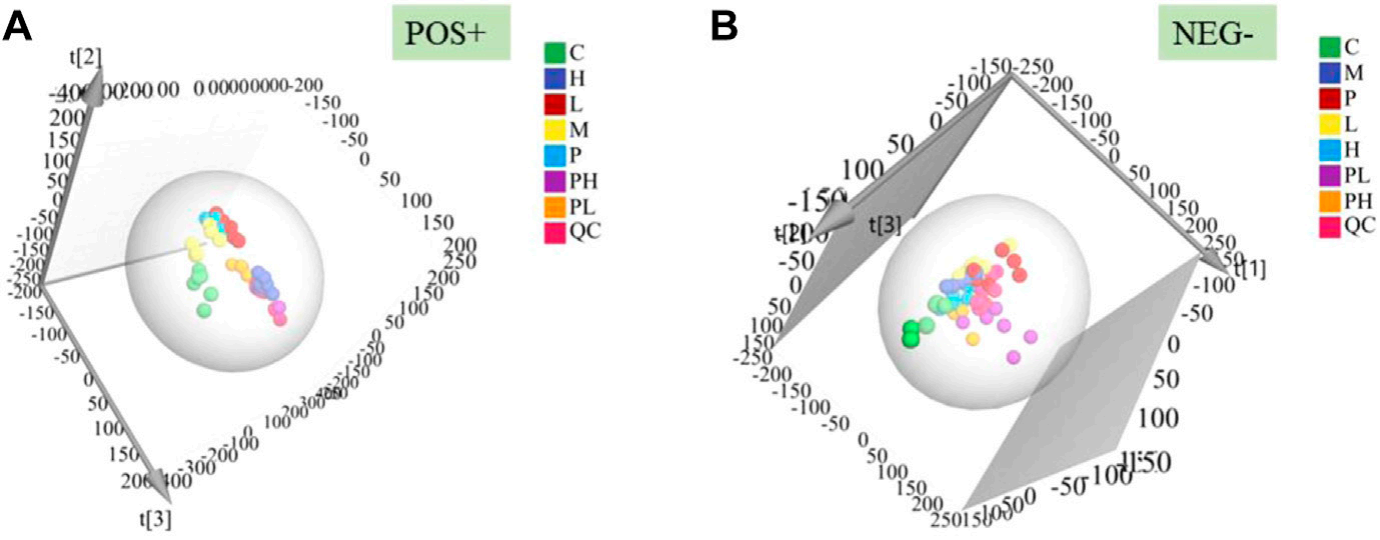
PCA score plots in positive **(A)** and negative **(B)** ion mode of serum samples among control [C], model [M], cisplatin [P], GYP low-dose [L], GYP high-dose [H], cisplatin + low-dose GYP [PL], cisplatin + high-dose GYP [pH] group and QC group [QC].

**FIGURE 5:**
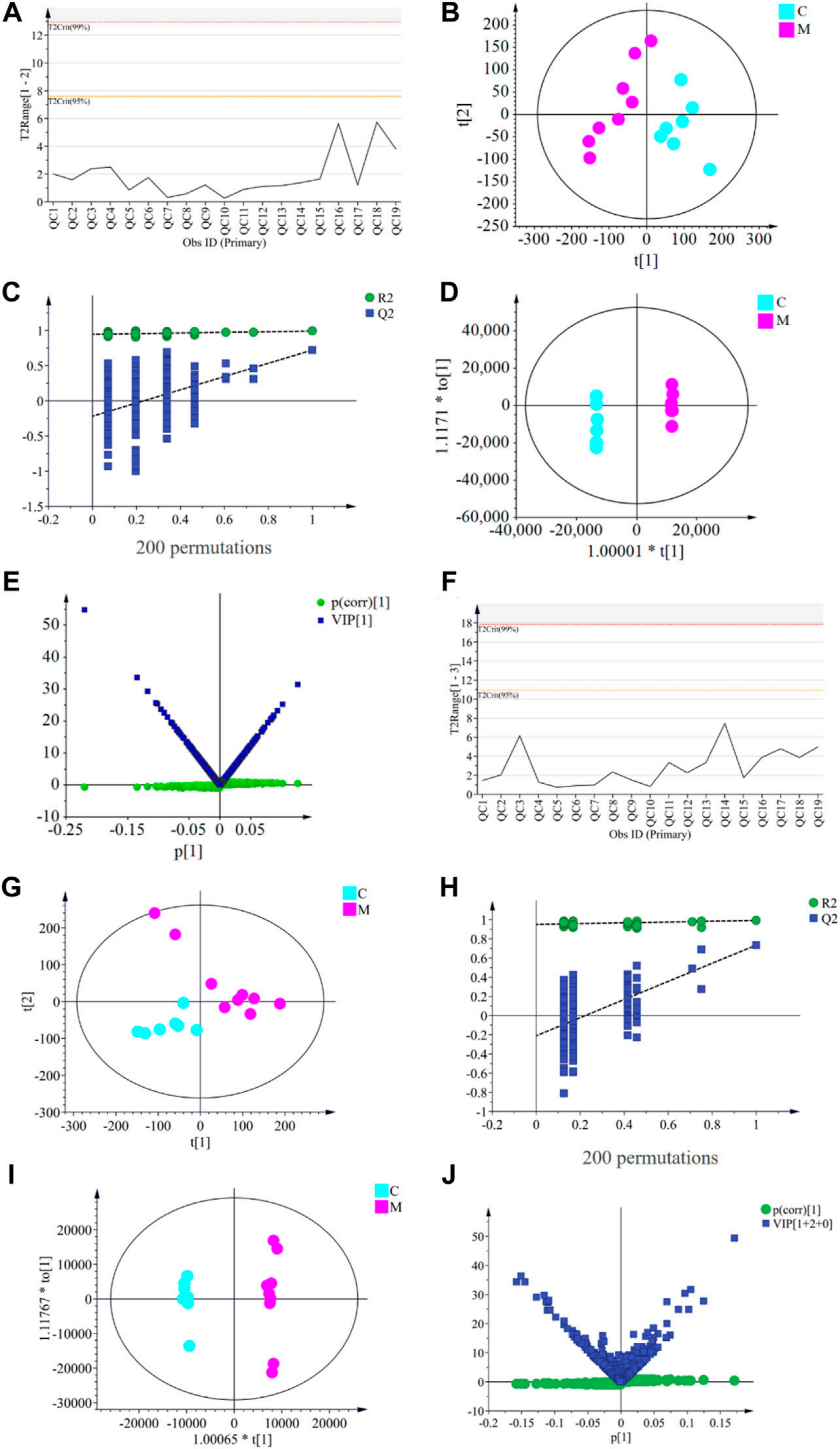
Hotelling’s T2 plots of QC group **(A** and **F)**; PCA **(B** and **G)**, validation plot **(C** and **H)**, OPLS-DA **(D** and **I)**, VIP + S plots **(E** and **J)** of serum samples between control [C] group and model [M] group in postive model **(A–E)** and negative model **(F–J)**.

The PCA score plots (Figures 4A,B) of 7 groups in two modes showed that GYP treatment had effect on the metabolic profile of LLC mice. According to Figures 5A,F, the Hotelling’s T2 range map showed that there were no serious outliers in all samples in the QC group, indicating the reliability and accuracy of the analysis. The PCA score plots (Figures 5B,G) and OPLS-DA score plots (Figures 5C,H) demonstrated that the C group and M group were well separated, which proved that the Lewis lung cancer mouse model is successful. The OPLS-DA model validation plots showed that R2 and Q2 values of all points on the left were less than those of the rightmost point, which proves that the model was successful (Figures 5D,I). Then VIP + S-plot scatter plots (Figures 5E,J) were used to seek the differential metabolites between the C group and the M group, then 53 differential biomarkers (Table 1) of LLC mice were identified by HMDB database, METLIN database and Analyst®TF 1.6 Software in positive and negative datasets.

**TABLE 1:** Identified differential metabolites in serum between control group [C] and model group [M] in positive ion model and negative ion model.

Name	Molecular formula	Mode	RT/min	ppm	VIP	*p*corr-value	*p*-value
Aminopropylcadaverine	C_8_H_21_N_3_	M + H	1.60	−1.87	1.15	0.71	<0.001
Choline	C_5_H_14_NO	M + H	1.88	−1.92	2.11	−0.60	0.0010
Taurine	C_2_H_7_NO_3_S	M - H	1.95	0.81	1.04	−0.79	0.0155
Formiminoglutamic acid	C_6_H_10_N_2_O_4_	M + H	2.39	−2.28	1.29	−0.92	<0.001
Cytosine	C_4_H_5_N_3_O	M + H	2.47	−2.68	1.20	−0.81	<0.001
Cytidine	C_9_H_13_N_3_O_5_	M + H	2.47	0.41	1.18	−0.74	<0.001
Indole-3-acetamide	C_10_H_10_N_2_O	M + H	2.48	−4.57	1.24	−0.90	<0.001
Pseudouridine	C_9_H_12_N_2_O_6_	M - H	2.50	2.89	2.37	0.77	0.0026
4-(2-Aminophenyl)-2,4-dioxobutanoic acid	C_10_H_9_NO_4_	M - H	5.40	1.46	1.12	−0.60	0.0108
N-Acetyl-L-methionine	C_7_H_13_NO_3_S	M - H	5.43	−4.21	1.81	−0.93	<0.001
Undecanedioic acid	C_11_H_20_O_4_	M - H	5.66	1.39	1.24	−0.69	<0.001
Butenylcarnitine	C_11_H_19_NO_4_	M - H	6.01	1.32	1.66	−0.68	<0.001
Indoleacetaldehyde	C_10_H_9_NO	M - H	6.25	0.63	1.05	0.72	<0.001
Tetradecanedioic acid	C_14_H_26_O_4_	M - H	7.33	0.78	5.65	−0.68	<0.001
3, 5-Tetradecadiencarnitine	C_21_H_37_NO_4_	M + H	10.51	−1.08	1.09	−0.66	<0.001
Dodecanoylcarnitine	C_19_H_38_NO_4_	M + H	10.65	−0.87	1.13	−0.65	0.0020
Pimelylcarnitine	C_14_H_25_NO_6_	M + H	12.02	−1.32	1.09	−0.73	0.0110
Sphingosine	C_18_H_37_NO_2_	M + H	12.50	−1.00	1.88	−0.66	<0.001
3-Oxododecanoic acid	C_12_H_22_O_3_	M - H	12.61	2.35	2.52	−0.58	<0.001
10,11-dihydro-20-trihydroxy-leukotriene B4	C_20_H_34_O_7_	M - H	13.34	2.60	9.06	−0.60	<0.001
Sphingosine 1-phosphate	C_18_H_38_NO_5_P	M + H	13.74	−0.79	2.45	0.72	<0.001
Dopamine 4-sulfate	C_8_H_11_NO_5_S	M + Na	14.07	4.30	2.88	−0.62	<0.001
12-Hydroxydodecanoic acid	C_12_H_24_O_3_	M - H	14.32	1.86	2.16	−0.65	<0.001
Sphinganine 1-phosphate	C_18_H_40_NO_5_P	M + H	14.41	−2.09	1.38	0.68	<0.001
LysoPA (8:0/0:0)	C_11_H_23_O_7_P	M + H	14.61	2.67	1.01	−0.60	0.0018
LysoPC(18:3 (6Z,9Z,12Z)/0:0)	C_26_H_48_NO_7_P	M + H	14.80	1.35	12.14	−0.69	<0.001
12S-HHT	C_17_H_28_O_3_	M - H	15.07	1.07	3.26	−0.77	<0.001
LysoPE (18:0/0:0)	C_23_H_48_NO_7_P	M + H	15.16	−1.04	6.65	−0.77	0.0176
VPGPR Enterostatin	C_23_H_40_N_8_O_6_	M + Na	15.66	4.36	4.23	−0.66	<0.001
LysoPC(15:0/0:0)	C_23_H_48_NO_7_P	M + H	15.79	0.21	6.65	−0.74	0.0160
Taurodeoxycholic acid	C_26_H_45_NO_6_S	M - H	15.95	0.60	2.31	0.51	<0.001
LysoPC(20:0/0:0)	C_28_H_58_NO_7_P	M + H	17.33	−1.63	4.52	−0.63	<0.001
Stearoylcarnitine	C_25_H_49_NO_4_	M + H	17.34	−0.93	5.02	0.93	<0.001
LysoPC(20:3 (8Z,11Z,14Z)/0:0)	C_28_H_52_NO_7_P	M - H	17.63	2.39	2.69	−0.72	<0.001
LysoPC(22:6 (4Z,7Z,10Z,13Z,16Z,19Z)/0:0)	C_30_H_50_NO_7_P	M + H	17.75	−4.75	1.07	0.59	<0.001
LysoPE (P-16:0/0:0)	C_21_H_44_NO_6_P	M - H	18.26	0.69	2.36	−0.70	<0.001
PI(18:0/18:0)	C_45_H_87_O_13_P	M - H	18.44	2.20	3.93	−0.65	<0.001
LysoPE (20:2 (11Z,14Z)/0:0)	C_25_H_48_NO_7_P	M - H	19.05	1.19	2.57	0.80	<0.001
LysoPC(20:2 (11Z,14Z)/0:0)	C_28_H_54_NO_7_P	M + H	19.40	−1.46	3.98	0.70	<0.001
LysoPC(P-18:0/0:0)	C_26_H_54_NO_6_P	M + H	19.49	−0.39	8.12	−0.78	<0.001
LysoPE (0:0/22:1 (13Z))	C_27_H_54_NO_7_P	M + H	20.20	−1.49	3.11	0.53	0.0037
Stearidonic acid	C_18_H_28_O_2_	M - H	21.36	2.18	4.69	−0.85	<0.001
Oleamide	C_18_H_35_NO	M + H	22.60	−1.06	1.31	−0.52	0.0034
2-Hexaprenyl-3-methyl-5-hydroxy-6-methoxy-1,4-benzoquinol	C_38_H_58_O_4_	M - H	22.94				<0.001
Tetradecanoylcarnitine	C_21_H_42_NO_4_	M + H	23.10	−1.07	3.74	0.66	<0.001
Oleoylethanolamide	C_20_H_39_NO_2_	M + H	23.26	−1.84	1.09	−0.56	0.0207
Guanosine triphosphate adenosine	C_20_H_27_N_10_O_17_P_3_	M + Na	25.79	2.39	3.12	0.78	<0.001
MG (14:0/0:0/0:0)	C_17_H_34_O_4_	M - H	26.38	1.00	2.66	−0.77	<0.001
LysoPC(6:0/0:0)	C_14_H_30_NO_7_P	M - H	26.38	−4.24	1.01	−0.69	<0.001
Cholesterol sulfate	C_27_H_46_O_4_S	M - H	26.72	0.64	3.51	−0.65	<0.001
Androsterone sulfate	C_19_H_30_O_5_S	M - H	27.66	4.33	5.00	−0.75	<0.001
13′-Hydroxy-alpha-tocopherol	C_29_H_50_O_3_	M + Na	28.09	−1.92	4.18	−0.62	<0.001
12,13-DHOME	C_18_H_34_O_4_	M + Na	31.88	−2.97	1.03	0.62	<0.001

**FIGURE 6:**
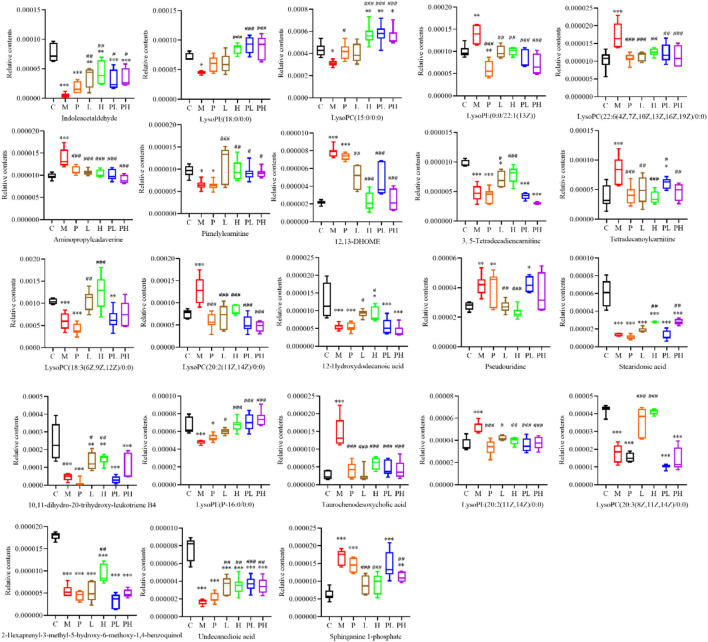
GYP significantly recovered the levels of 23 differential metabolites. Data were analyzed by One-way ANOVA analysis and represented as means ± SD (n = 6). (C: control group, M: model group, P: cisplatin group, L: low-dose GYP group, H: high-dose GYP group, PL: cisplatin + low-dose GYP group, PH: cisplatin + high-dose GYP group; vs. Control group, *p < 0.05, **p < 0.01, ***p < 0.001; vs. Model group, ^#^ *p* < 0.05, ^##^ *p* < 0.01, ^###^ *p* < 0.001).

The relative contents of 53 differential metabolites among 7 groups (including control [C], model [M], cisplatin [P], GYP low-dose [L], GYP high-dose [H], cisplatin + low-dose GYP [PL] and cisplatin + high-dose GYP [pH]) was compared by GraphPad Prim 9 software (Supplementary Figure S1). As shown in Figure 6, GYP could regulate the metabolic disorder caused by LLC, a total of 23 metabolites had a tendency to recover to the C group level, in other words, a total of 23 potential metabolites in GYP low-dose [L] and GYP high-dose [H] group could be recalled to the content close to the C group compared with M group, including indoleacetaldehyde, LysoPE (18:0/0:0), LysoPC(15:0/0:0), LysoPE (0:0/22:1 (13Z)), LysoPC(22:6 (4Z,7Z,10Z,13Z,16Z,19Z)/0:0), aminopropylcadaverine, pimelylcarnitine, 12,13-DHOME, 3,5-Tetradecadiencarnitine, tetradecanoylcarnitine, LysoPC(18:3 (6Z,9Z,12Z)/0:0), LysoPC(20:2 (11Z,14Z)/0:0), 12-Hydroxydodecanoic acid, pseudouridine, stearidonic acid, 10,11-dihydro-20-trihydroxy-leukotriene B4, LysoPE (P-16:0/0:0), taurochenodesoxycholic acid, LysoPE (20:2 (11Z,14Z)/0:0), LysoPC(20:3 (8Z,11Z,14Z)/0:0), 2-Hexaprenyl-3-methyl-5-hydroxy-6-methoxy-1,4-benzoquinol, Undecanedioic acid, sphinganine 1-phosphate.

### 3.4 Metabolic pathway analysis of 23 metabolites associated with GYP treatment

**FIGURE 7:**
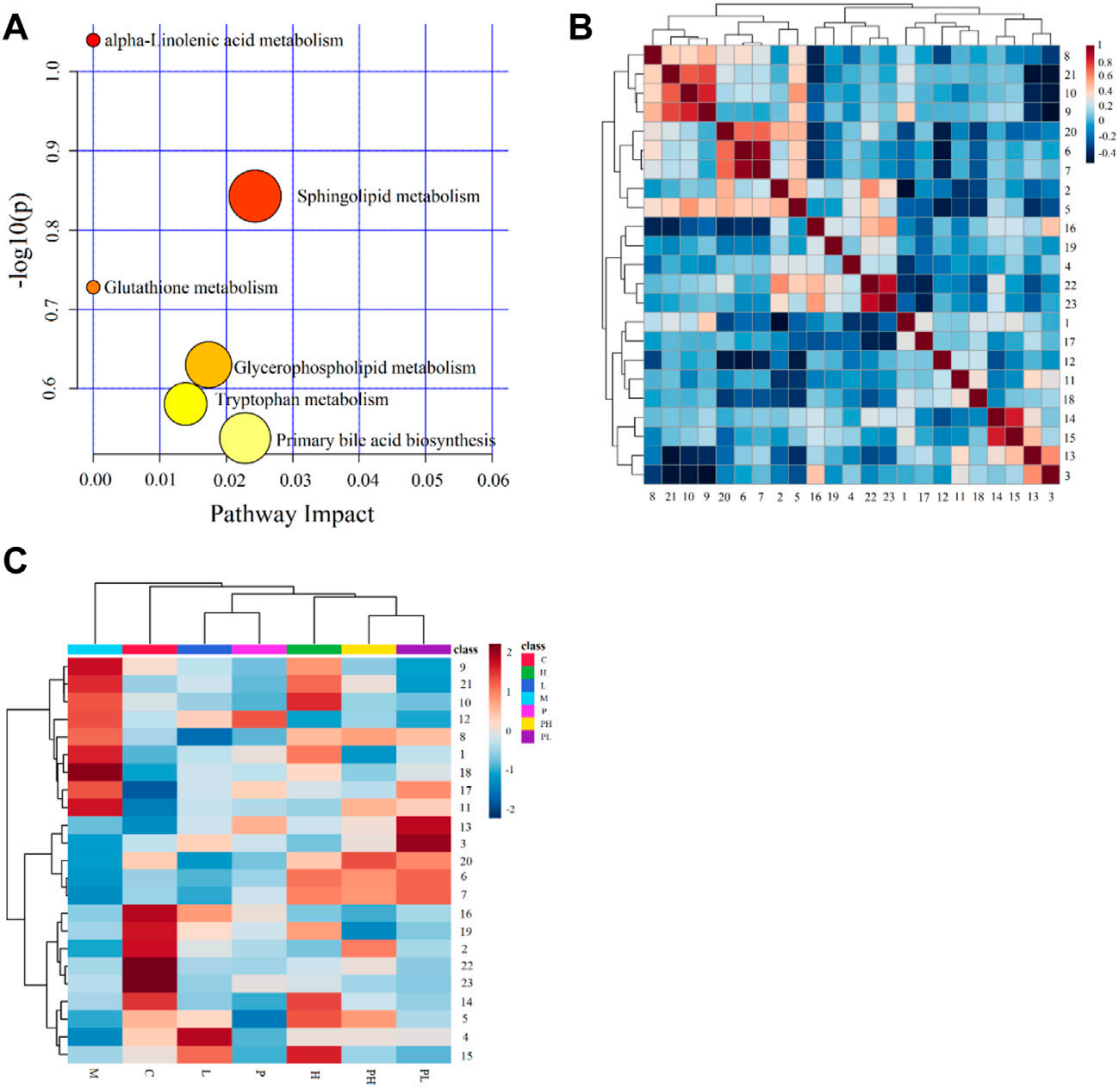
Pathway analysis of 23 differential metabolites of GYP on LLC mice **(A)**; Pearson correlation analysis results of 23 differential metabolites, red indicates positive correlation and blue indicates negative correlation **(B)**; Heatmap analysis of 23 differential metabolites among 7 groups **(C)**. (Metabolites 1-23 represent Aminopropylcadaverine, Indoleacetaldehyde, 3,5-Tetradecadiencarnitine, Pimelylcarnitine, LysoPC [18:3 (6Z,9Z,12Z)/0:0], LysoPE (18:0/0:0), LysoPC (15:0/0:0), LysoPC [22:6 (4Z,7Z,10Z,13Z,16Z,19Z)/0:0], LysoPC [20:2 (11Z,14Z)/0:0], LysoPE [0:0/22:1 (13Z)], Tetradecanoylcarnitine, 12,13-DHOME, Pseudouridine, Undecanedioic acid, 10,11-dihydro-20-trihydroxy-leukotriene B4, 12-Hydroxydodecanoic acid, Sphinganine 1-phosphate, Taurochenodesoxycholic acid, LysoPC [20:3 (8Z,11Z,14Z)/0:0], LysoPE (P-16:0/0:0), LysoPE [20:2 (11Z,14Z)/0:0], Stearidonic acid, 2-Hexaprenyl-3-methyl-5-hydroxy-6-methoxy-1,4-benzoquinol).

For exploring the effect of GYP on Lewis tumor-bearing mice, 23 metabolites of GYP on LLC mice were introduced into MetaboAnalyst 5.0 database to select *Mus musculus* (KEGG) for path Library for metabolic pathway analysis. According to Figure 7A, we could judge that the effect of GYP on Lewis tumor-bearing mice may be related to six metabolic pathways, including alpha-Linolenic acid metabolism, glutathione metabolism, sphingolipid metabolism, glycerophospholipid metabolism, tryptophan metabolism, primary bile acid biosynthesis. The metabolite involved in alpha-Linolenic acid metabolism was stearidonic acid; The metabolite related to sphingolipid metabolism was sphinganine 1-phosphate; Aminopropylcadaverine was related to glutathione metabolism; The metaboletes involved in glycerophospholipid metabolism was LysoPC(15:0/0:0), LysoPC(22:6 (4Z,7Z,10Z,13Z,16Z,19Z)/0:0), LysoPC(18:3 (6Z,9Z,12Z)/0:0), LysoPC(20:2 (11Z,14Z)/0:0); Indoleacetaldehyde was involved in tryptophan metabolism; The metabolite related to primary bile acid biosynthesis was taurochenodesoxycholic acid.

### 3.5 Pearson correlation analysis and heatmap analysis

Pearson correlation analysis (Figure 7B) was used to study the correlation between 23 different metabolites, in which there was a strong positive correlation between undecanedioic acid and 10,11-dihydro-20-trihydroxy-leukotriene B4, stearidonic acid and Hexaprenyl-3-methyl-5-hydroxy-6-methoxy-1,4-benzoquinol, LysoPE (18:0/0:0) and LysoPC(15:0/0:0). There was a strong negative correlation between these metabolites, such as 3, 5-Tetradecadiencarnitine and LysoPC(20:2 (11Z,14Z)/0:0), pseudouridine and LysoPE (20:2 (11Z,14Z)/0:0). These results suggest that these differential metabolites were interrelated. The heatmap analysis (Figure 7C) was used to analyze the relative content of 23 metabolites in 7 groups.

### 3.6 Construction of a metabolite-target network

**FIGURE 8:**
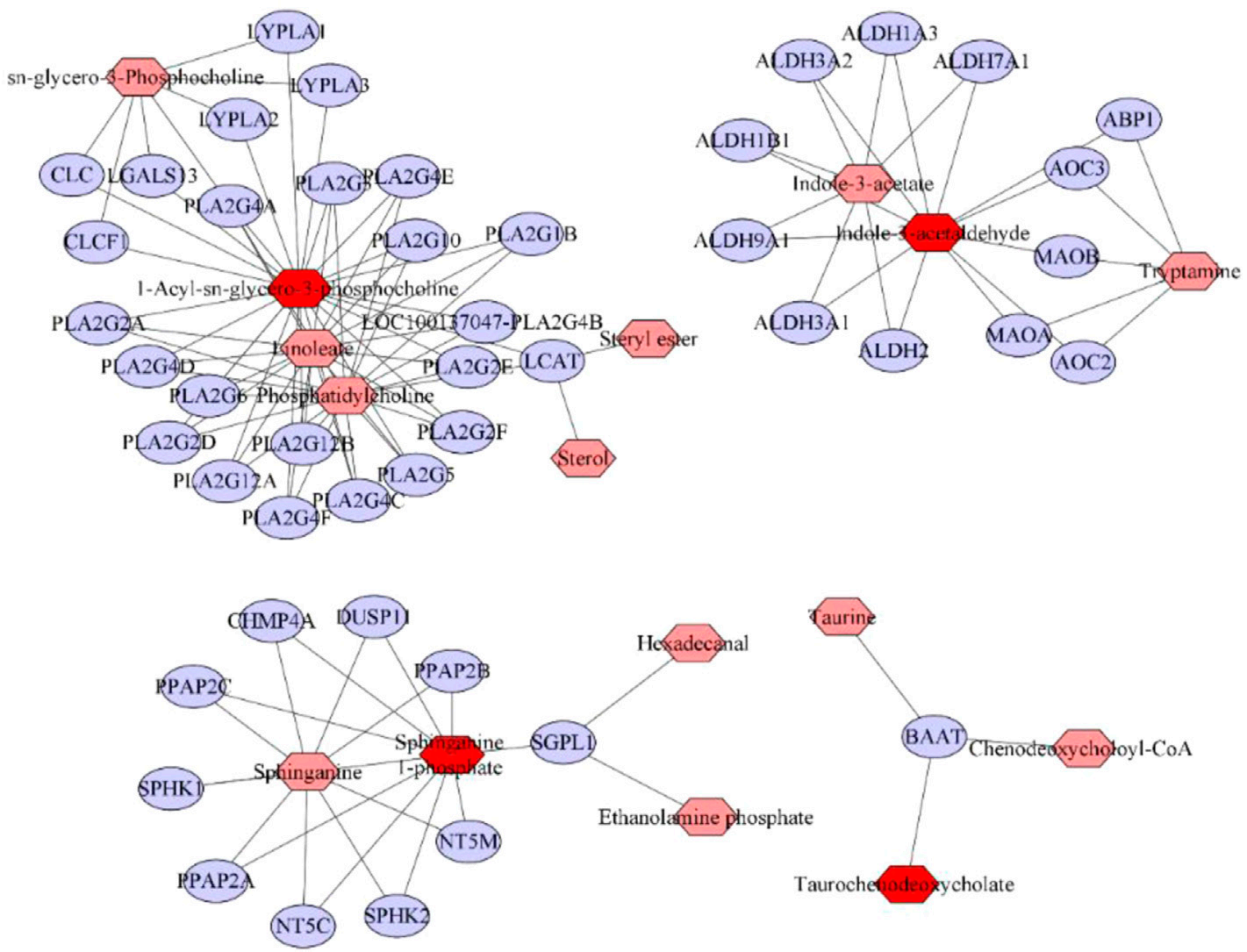
Metabolite-Gene network of GYP on LLC mice. Red, pink and blue nodes respectively represent differential metabolites of GYP recovery, interacting metabolites and metabolite related gene.

The KEGG ID of metabolites of GYP on lung cancer was introduced into MetScape (a plug-in of Cytoscape) to build the metabolite-target network (Figure 8). The related genes of LysoPC(15:0/0:0), LysoPC(22:6 (4Z,7Z,10Z,13Z,16Z,19Z)/0:0), LysoPC(18:3 (6Z,9Z,12Z)/0:0), LysoPC(20:2 (11Z,14Z)/0:0) were respectively LYPLA1, LYPLA2, CLC, PLA2G4E, CLCF1, LYPLA3, PLA2G4F, PLA2G2D, PLA2G4D, LGALS13, PLA2G2E, LCAT, PLA2G3, PLA2G1B, PLA2G2A, PLA2G4A, PLA2G5, PLA2G2F, PLA2G12A, PLA2G6, PLA2G10, PLA2G12B, PLA2G4C, LOC100137047-PLA2G4B; Sphinganine 1-phosphate related genes included CHMP4A, NT5C, SPHK2, NT5M, DUSP11, PPAP2A, PPAP2C, PPAP2B, SPHK1, SGPL1; Indoleacetaldehyde related genes included ALDH2, ALDH3A1, ALDH1B1, ALDH1A3, ALDH9A1, ALDH3A2, ABP1, AOC2, MAOA, MAOB, ALDH7A1, AOC3; Taurochenodeoxycholate related gene was BAAT. A total of 57 metabolite-related genes were obtained for the subsequent analysis.

### 3.7 Network analysis

**FIGURE 9:**
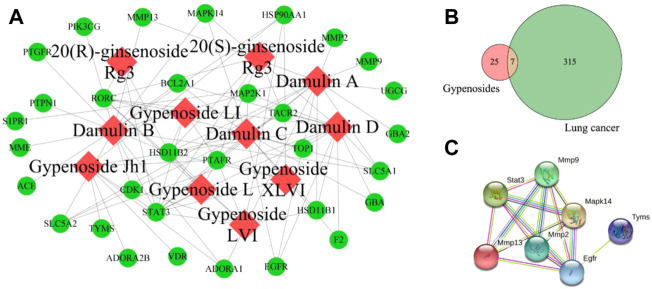
The compounds-target network of GYP on lung cancer, the red nodes and green nodes represent 11 saponin components and genes **(A)**; Wayne diagram of intersection gene between GYP and lung cancer **(B)**; PPI network for GYP against lung cancer **(C)**.

In our previous report, 11 saponin components in GYP were identified by HPLC-MS-IT-TOF Network pharmacology analysis (Qi et al., 2021). Then the compound-target network between 11 saponins and their related genes was constructed (Figure 9A), this network includes 11 components and 32 target genes. 32 GYP related genes were retrieved from SwissTargetPrediction database, 322 LC related targets were retrieved from NCBI-gene database (https://www.ncbi.nlm.nih.gov/gene). There are 7 intersection genes of 11 components and diseases, namely STAT3, MMP9, MMP2, MMP13, EGFR, TYMS, MAPK14 (Figures 9B,C).

### 3.8 Multi network construction

**FIGURE 10:**
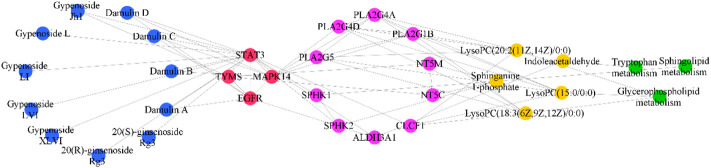
Compound-intersection gene-metabolite related gene-metabolites-pathway network. Blue nodes, red nodes, purple nodes, yellow nodes, and green nodes represented 11 components of GYP, intersection targets of components and diseases, metabolite related targets, differential metabolites of GYP recovery, and metabolic pathways, respectively.

The compound-intersection genes-metabolite related gene-metabolite-pathway network (Figure 10) showed that 11 saponins of GYP might indirectly regulate LysoPC (15:0/0:0), LysoPC (22:6 (4Z, 7Z, 10Z, 13Z, 16Z, 19Z)/0:0), LysoPC (18:3 (6Z, 9Z, 12Z)/0:0), LysoPC (20:2 (11Z, 14Z)/0:0), indoleacetaldehyde, sphinganine 1-phosphate and then affect glycerophospholipid metabolism, sphingolipid metabolism, tryptophan metabolism to play an anticancer effect.

### 3.9 Validation of critical targets

**FIGURE 11:**
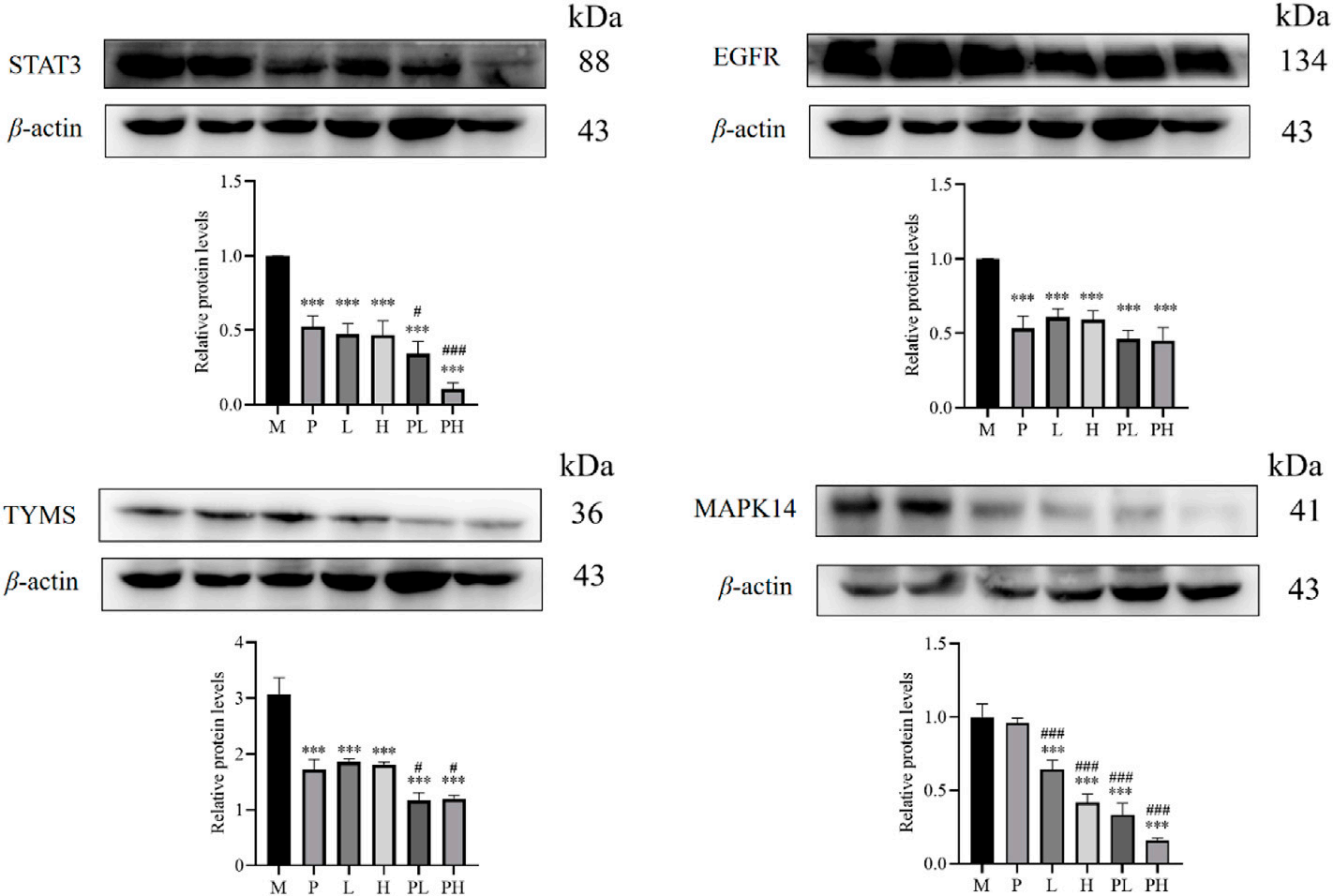
The effect of GYP and/or cisplatin on the protein levels of STAT3, EGFR, TYMS and MAPK14 in tumor tissues among M, P, L, H, PL and PH group. (M: model group; P: cisplatin group, L: low-dose GYP group, H: high-dose GYP group, PL: cisplatin + low-dose GYP group, PH: cisplatin + high-dose GYP group; vs. model group, *p < 0.05, **p < 0.01, ***p < 0.001; vs. cisplatin group, ^#^ *p* < 0.05, ^##^ *p* < 0.01, ^###^ *p* < 0.001).

In order to investigate how GYP exerts its anti-LC effects by regulating metabolites and metabolic pathways as well as enhancing the anti-lung cancer effects of chemotherapeutic agents, the relative contents of STAT3, EGFR, MAPK14, and TYMS between the six groups were analyzed by western blot (Figure 11). MAPK14 and STAT3 play a carcinogenic role in the research mechanism of cancer cells (Yang et al., 2021). As shown in Figure 11, GYP group significantly inhibited the expression of STAT3, EGFR, TYMS and MAPK14 in tumor tissues compared with M group, the expression of STAT3, TYMS and MAPK14 in the GYP + cisplatin group ([PL] and [PH]) were significantly lower than that in the model group [M], single administration group ([P], [L] and [H] group). The findings suggested that GYP might exert anticancer effects in lung cancer and enhance the effects of chemotherapeutic agent cisplatin through the MAPK14/STAT3 signaling pathway.

## 4 Discussion

Our study showed that GYP could inhibit the proliferation of LLC cells and significantly reduced the tumor weight and tumor volume of LLC mice, and the combination of GYP and cisplatin had a better effect than cisplatin alone and GYP alone. Histopathological studies showed that GYP could promote apoptosis and necrosis of tumor cells, and the combination of GYP and cisplatin was more effective than GYP alone. UPLC-Q-TOF/MS was used to investigate the mechanism of GYP inhibition of lung cancer and enhancement of cisplatin anticancer effect from the perspective of endogenous metabolites. Serum metabolomics identified 53 biomarkers related to LLC mice, of which GYP was able to significantly recover 23 differential metabolites, and the effect of GYP on LLC mice might be related to six metabolic pathways, including alpha-Linolenic acid metabolism, glutathione metabolism, sphingolipid metabolism, glycerophospholipid metabolism, tryptophan metabolism, primary bile acid biosynthesis. Network analysis and metabolomics combined with western blot demonstrated that GYP might exert anti-lung cancer effects as well as enhance the pharmacological effects of the chemotherapeutic agent cisplatin through the MAPK14/STAT3 signaling pathway.

In our study, the relative contents of 3,5-tetradecadienoic acid, 12-hydroxydodecanoic acid and indole-3 acetaldehyde in the serum of mice in the model group decreased, while GYP increased the levels of these metabolites; The relative contents of 12,13-DHOME, pseudouridine, sphinganine 1-phosphate and taurochenodesoxycholic acid in the serum of LLC mice in the model group was increased, but GYP decreased their levels. 3,5-tetradecadiencarnitine is one of the metabolic biomarkers of thyroid papillary carcinoma in different iodine nutrition regions, and it is related to iodine excess and cancer (Sun et al., 2021). Studies have shown that 12,13-DHOME comes from the oxidation of linoleic acid, which is related to an increased risk of ovarian cancer, cytochrome P450 monooxygenase oxidizes linoleic acid to produce 12,13-DHOME, the role of DHOME in multiple cellular functions may lead to an increased risk of ovarian cancer (Hada et al., 2019). 12-Hydroxydodecanoic acid is one of the biomarkers of serum metabolites of colorectal cancer (Zhang et al., 2021). Indole-3 acetaldehyde is a precursor molecule for tryptophan to form endogenous 6-formylindolo [3,2-b] carbazole (ficz), tryptophan is related to tumor immune escape (Rannug, 2022). Pyridyl carnitine is one of the potential biomarkers for early diagnosis of hepatecellular carcinoma (HCC) (Kim et al., 2019). Pseudouridine was disturbed in patients with epithelial ovarian cancer (EOC), which might be a potential biomarker of EOC (Zhang et al., 2013). Sphinganine 1-phosphate (S1P) is one of the plasma biomarkers in patients with esophageal squamous cell carcinoma (ESCC), which can be used to diagnose ESCC, S1P could promote inflammation and mitosis, which may help to promote the progress of pancreatic cancer, S1P acts on specific G protein coupled receptors on immune, cardiovascular and nervous system cells, thereby regulating the induction of inflammation and cancer (Scherer et al., 2009). Taurochenodesoxycholic acid is a potential biomarker associated with liver cirrhosis (Yin et al., 2009).

GYP significantly reduced the expression of STAT3, EGFR, TYMS and MAPK14 in tumor tissues, while cisplatin combined with GYP could further reduce the expression of STAT3, TYMS and MAPK14 in tumor tissues. MAPK14 (also known as p38 MAPK) plays a crucial role in many processes, such as cell survival, differentiation (Liang et al., 2015). As a member of MAPK family, MAPK14 plays a vital role in the regulation of apoptosis, MAPK14 is also overexpressed in breast, ovary and cancer cells to promote tumorigenesis and development. STAT3 signal could promote the growth of non-small cell lung cancer (NSCLC) by promoting angiogenesis, cell survival, cancer cell stem cells, so STAT3 could affect tumorigenesis by controlling the survival, apoptosis and cell cycle of cancer cells, the activation of STAT3 can promote the secretion of proinflammatory cytokines, growth factors and chemokines (Parakh et al., 2021). The expression level of TYMS in colorectal cancer patients was significantly higher than that in normal control group (Jie et al., 2021). TYMS is a biomarker of pancreatic cancer, which is up-regulated in pancreatic cancer (Fu et al., 2019). Epidermal growth factor receptor (EGFR) is a carcinogen and activates various carcinogenic signaling pathways in cancer cells (Yang et al., 2022). Overexpression and activation of EGFR promote cell growth and metastasis of many cancers (Zhang et al., 2022). EGFR, as a receptor tyrosine kinase (RTK), is a carcinogen and a therapeutic target for many cancers, which can activate many carcinogenic signal pathways in cancer cells.

Studies have shown that tryptophan metabolism is significantly increased in tumors, which can promote tumorigenesis and immune escape (Cui et al., 2021). It is reported that there is a correlation between glycerophospholipids metabolic disorder and tumorigenesis, and glycerophospholipids are associated with tumorigenesis and development, glycerophosphatides are the main components of biofilms (Chen et al., 2022), glycerolphospholipid metabolism is the most critical pathway associated with NSCLC (Wu et al., 2020). Lung cancer can cause regulation of sphingolipid metabolism, GYP can regulate this sphingolipid metabolism. Sphingolipids can effectively inhibit tumor formation (Lee et al., 2020). Sphingomyelin is a kind of sphingolipids, sphingolipids are key components of biofilms and participate in many processes related to tumor progression (Martin-Blazquez et al., 2019). However, there are still some deficiencies in this study, how GYP indirectly affect metabolites and metabolic pathways by affecting MAPK14/STAT3 signaling pathway still needs further research, we will carry out relevant research in the next step.

## 5 Conclusion

From the perspective of metabolomics and network pharmacology, we studied the mechanism of GYP on lung cancer and its mechanism of enhancing the anti-lung cancer pharmacological effects of cisplatin, GYP might exert anticancer effects as well as enhance the anticancer effects of cisplatin through the MAPK14/STAT3 signaling pathway, which provides a reference basis for the development of lung cancer drugs.

## Data Availability

The datasets presented in this study can be found in online repositories. The names of the repository/repositories and accession number(s) can be found in the article/Supplementary Material.

## Abbreviations

GYP, Gypenosides; LC, Lung cancer; MTT, 3- (4,5)-dimethylthiahiazo (-z-y1)-3,5-di- phenytetrazoliumromide; OD, Optical density; LLC, Lewis lung carcinoma cell; RT, Retention time; SDS-PAGE, Sodium dodecyl sulfate polyacrylamide gel electrophoresis; EP, eppendorf; QC, Quality control; TICs, Total ion chromatograms; VIP, Variable important in projection; STAT3, Signal Transducer and Activator of Transcription 3; TYMS, Thymidylate synthase; MAPK14, Mitogen-activated protein kinase 14; EGFR, Epidermal Growth Factor Receptor.
