# Supplementary material for: Comprehensive serum metabolomics and network analysis to reveal the mechanism of gypenosides in treating lung cancer and enhancing the pharmacological effects of cisplatin
Source: Front Pharmacol. 2022 Dec 1;13:1070948. doi: 10.3389/fphar.2022.1070948 (PMC9751056; doi:10.3389/fphar.2022.1070948)
Supplement: Supplementary file 1 [file Image1.pdf]

frontiers

Supplementary Material

1.1 Supplementary Figure

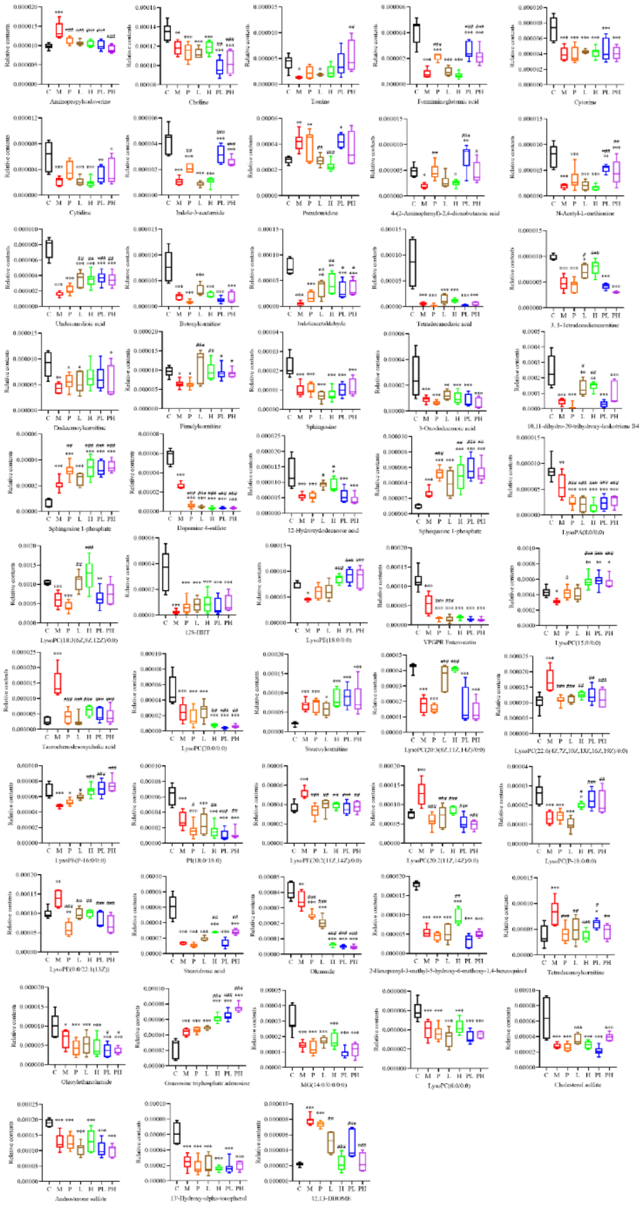

Supplementary Figure 1. The relative contents of 53 differential metabolites among 7 groups. Data were analyzed by One-way ANOVA analysis and represented as means ± SD (n = 6). (C: control group, M: model group, P: cisplatin group, L: low-dose GYP group, H: high-dose GYP group, PL: cisplatin + low-dose GYP group, PH: cisplatin + high-dose GYP group; *vs*. Control group, $^{*}p < 0.05$, $^{**}p < 0.01$, $^{***}p < 0.001$; *vs*. Model group, $^{\#}p < 0.05$, $^{\#\#}p < 0.01$, $^{\#\#\#}p < 0.001$).
